# The frequent evolutionary birth and death of functional promoters in mouse and human

**DOI:** 10.1101/gr.190546.115

**Published:** 2015-10

**Authors:** Robert S. Young, Yoshihide Hayashizaki, Robin Andersson, Albin Sandelin, Hideya Kawaji, Masayoshi Itoh, Timo Lassmann, Piero Carninci, The FANTOM Consortium, Wendy A. Bickmore, Alistair R. Forrest, Martin S. Taylor

**Affiliations:** 1 MRC Human Genetics Unit, MRC Institute for Genetics and Molecular Medicine, University of Edinburgh, Edinburgh, EH4 2XU, United Kingdom;; 2 RIKEN Preventive Medicine and Diagnosis Innovation Program, Wako, Saitama, 351-0198, Japan;; 3 Department of Biology and Biotech Research and Innovation Centre, Copenhagen University, 2200 Copenhagen N, Denmark;; 4 RIKEN Center for Life Science Technologies, Division of Genomic Technologies, Tsurumi-ku, Yokohama, 230-0045, Japan;; 5 Systems Biology and Genomics, Harry Perkins Institute of Medical Research, QEII Medical Centre, Nedlands, Western Australia 6009, Australia

## Abstract

Promoters are central to the regulation of gene expression. Changes in gene regulation are thought to underlie much of the adaptive diversification between species and phenotypic variation within populations. In contrast to earlier work emphasizing the importance of enhancer evolution and subtle sequence changes at promoters, we show that dramatic changes such as the complete gain and loss (collectively, turnover) of functional promoters are common. Using quantitative measures of transcription initiation in both humans and mice across 52 matched tissues, we discriminate promoter sequence gains from losses and resolve the lineage of changes. We also identify expression divergence and functional turnover between orthologous promoters, finding only the latter is associated with local sequence changes. Promoter turnover has occurred at the majority (>56%) of protein-coding genes since humans and mice diverged. Tissue-restricted promoters are the most evolutionarily volatile where retrotransposition is an important, but not the sole, source of innovation. There is considerable heterogeneity of turnover rates between promoters in different tissues, but the consistency of these in both lineages suggests that the same biological systems are similarly inclined to transcriptional rewiring. The genes affected by promoter turnover show evidence of adaptive evolution. In mice, promoters are primarily lost through deletion of the promoter containing sequence, whereas in humans, many promoters appear to be gradually decaying with weak transcriptional output and relaxed selective constraint. Our results suggest that promoter gain and loss is an important process in the evolutionary rewiring of gene regulation and may be a significant source of phenotypic diversification.

It has long been speculated that changes in transcriptional regulation underlie many of the phenotypic differences between species (King and Wilson 1975; Wittkopp and Kalay 2012), and there is abundant evidence for gene expression divergence between even closely related lineages (McCarroll et al. 2004; Khaitovich et al. 2005; Tirosh et al. 2006; Landry et al. 2007; De et al. 2009). Alterations in gene expression are also thought to be responsible for many of the traits segregating in populations (Munafo et al. 2003) and may contribute important somatic changes to the development of cancer (Ongen et al. 2014). Despite this considerable interest, we currently have limited insight into which DNA sequence changes impact transcriptional regulation or how such regulatory networks evolve (Stolfi et al. 2014).

The core promoter is the 150–200 nt of DNA on which the RNA polymerase II pre-initiation complex is assembled and where transcription initiates (Sandelin et al. 2007). Defining an active transcription start site (TSS) thus defines the downstream boundary of a functional core promoter. DNA sequence changes in core promoters represent obvious candidates for transcriptional regulatory changes, and expression quantitative trait loci (eQTLs) are enriched within or close to these regions (Dimas et al. 2009). Similarly, genome-wide associations for diverse traits are significantly enriched in and around core promoters (Kindt et al. 2013). In contrast to the importance of promoter sequence changes implied by population genetic studies, the few studies that have directly compared gene expression patterns between species found that expression divergence generally does not correlate well with sequence changes in the core promoter (Tirosh et al. 2006). In the case of a comparison between human and mouse macrophage stimulus response, promoter sequence divergence was significantly anti-correlated with expression divergence (Schroder et al. 2012).

In contrast to these studies’ focus on nucleotide substitution changes at orthologous sequence, evolution can also proceed through the complete gain or loss (we subsequently refer to gain and loss events collectively as turnover) of functional genetic elements. The frequent turnover of small, discrete transcription factor binding sites was a striking and initially surprising finding that is at least sometimes associated with changes in transcriptional regulation (Cotney et al. 2013; Ballester et al. 2014; Vierstra et al. 2014; Villar et al. 2014). Although transcription factor binding site gain and loss has been proposed as a means of regulatory diversification (Odom et al. 2007; Cheng et al. 2014), questions remain as to whether the majority of experimentally measured binding sites have an impact on transcriptional regulation, let alone organismal phenotypes. Similarly, instances of TSS turnover have been observed between mouse and human (Frith et al. 2006), showing that the transcriptional regulation of genes can also be dramatically modified by the gain or loss of a promoter, despite the common assumption that enhancers rather than promoters are the primary drivers of *cis*-regulatory divergence (Brown and Feder 2005; Wittkopp and Kalay 2012; Villar et al. 2015). We previously demonstrated that human TSSs often failed to align with orthologous regions in other mammalian genomes (Forrest et al. 2014) and that this was most evident for promoters with a restricted breadth of expression. As with other previous work (Frith et al. 2006), we were unable to discriminate gains from losses, or assign the change to either the mouse or human lineage.

Motivated by these initial observations of dramatic evolutionary changes, we have set out to discover the extent to which the gain, loss, and divergence of functional promoters contribute to regulatory evolution along both the rodent and human lineages. We have used extensive libraries of cap analysis of gene expression (CAGE) data, which precisely and quantitatively define transcription start sites (Forrest et al. 2014). The CAGE libraries were generated from a broad range of human and mouse samples, including 52 tissues and cell types that are matched between the two species. We have identified promoters whose sequence is conserved between species and those for which there is no orthologous sequence (sequence turnover). With reference to genome sequences from multiple outgroup species (horse, dog, cow, and pig), sequence turnovers were resolved into either insertions or deletions and the change assigned to either the human or rodent lineage. Where promoters do align between species, we compared expression across the 52 matched tissues to score the conservation of expression and contrasted this with measures of nucleotide substitution constraint.

## Results

### The evolutionary histories and fates of mammalian promoters

**Figure 1.**
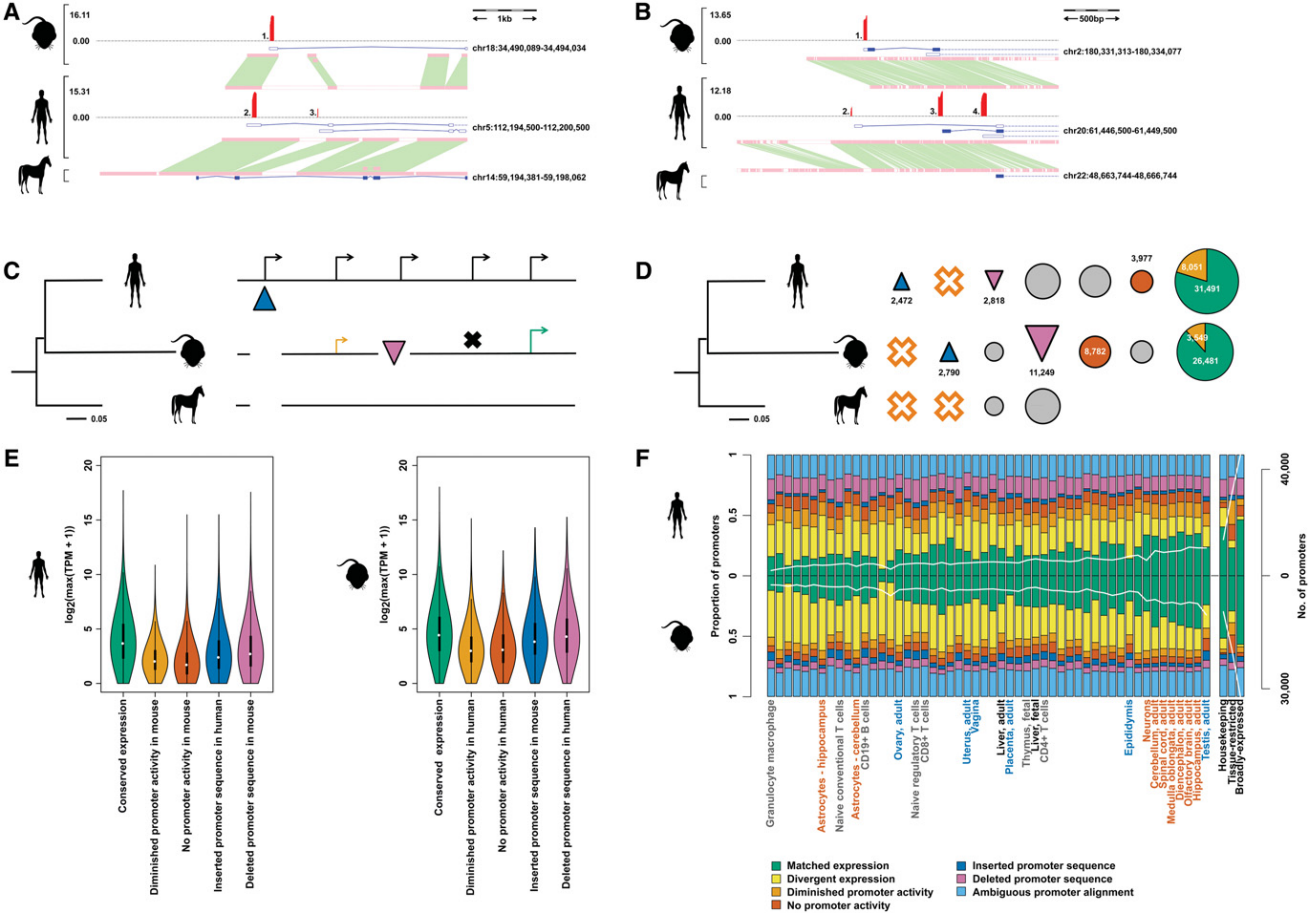
Evolutionary outcomes of human and mouse promoters. Horse is shown here as the example outgroup species, although promoters are identified as being present ancestrally if they are found in at least one, but not all, outgroup species (see Methods). (*A*,*B*) Example promoter insertions and deletions. Gene models supported by the CAGE promoters are shown in the blue boxes, where closed boxes represent coding exons and empty boxes noncoding exons. The histograms in red describe the log_2_-transformed expression level of the annotated promoters. Orthologous sequence identified between species is highlighted by the green boxes between these sequences. (*A*) Promoter insertion at the *SRP19* locus in the human lineage. Promoters 1 and 2 are conserved, while promoter 3 has been inserted in the human lineage. (*B*) Promoter deletion at the *Col9a3* locus in the mouse lineage. Promoters 1 and 3 are conserved, promoter 2 has been deleted in the mouse lineage, and promoter 4 has experienced expression turnover between human and mouse. (*C*) Schematic diagram showing each possible evolutionary fate of a human promoter. Promoters are denoted by the black arrows in human, where the blue triangle shows a recently inserted promoter in the human lineage and the purple triangle shows a recently deleted promoter in the mouse lineage. Aligned (black horizontal lines) promoters can show either matched (green arrow) or diminished (yellow arrow) expression in mouse. A human promoter which has completely lost its promoter ability in mouse is shown by the black cross. (*D*) Frequencies of inserted, deleted, aligned but no promoter activity (orange circles), or conserved (matched, divergent, and diminished) promoters in human and mouse. The lack of tissue-matched CAGE data from an outgroup species prevented us from assigning these expression changes to a specific lineage, so these events can only be classed as expression turnovers between human and mouse. The yellow segments in the conserved promoters show the proportion of promoters with diminished expression in the opposite species. (*E*) Maximum expression values for promoters with each evolutionary outcome as described and quantified in *D* in human (*left* panel) and mouse (*right* panel). (*F*) Proportion of promoters displaying each evolutionary outcome in human and mouse. Samples are ordered by rank of human:mouse average promoter count per sample. The white line denotes the number of promoters with that tissue bias or expression profile (*right* axis), and the frequencies of each evolutionary outcome for each tissue bias or expression profile are detailed in Supplemental Table 2. Tissues used in subsequent groupings (reproductive, blue; brain, orange; immunity, yellow) or mentioned directly in the text (liver) are labeled individually. This figure is reproduced as Supplemental Figure 1, where all tissues are labeled.

The genomic coordinates of CAGE-defined “robust” TSSs (Forrest et al. 2014) from human (*n* = 76,445) and mouse (*n* = 51,611) were projected into orthologous genomic positions between species using whole-genome multiple sequence alignments (see Methods). Since core promoters do not have a readily definable upstream boundary and typically exhibit heterogeneity in their precise site of transcription initiation (Carninci et al. 2006), we have used the genomic span of the CAGE tag-defined TSS cluster as a proxy for the promoter. A human promoter projected into mouse sequence was considered aligned. Its projection into an alignment gap indicates either de novo insertion in the human lineage or deletion from the mouse lineage (sequence turnover). These two possibilities were resolved by reference to alignment with four outgroup species (dog, horse, cow, and pig). An insertion in the human lineage (Fig. 1A, promoter 3) will be missing from the alignments with all outgroup species, while alignment with any of these suggests the promoter-containing sequence was present in the human:mouse ancestor and so represents a deletion on the mouse lineage (Fig. 1B, promoter 2).

We find 2472 de novo insertions of promoter-containing sequence in the human lineage and marginally more (2818) human lineage deletions (Fig. 1D). This represents turnover within the human lineage of ∼10% of the extant human promoters for which we can confidently infer the evolutionary history. In the mouse lineage, we find 2790 de novo insertions of functional promoters and 11,249 deletions. This is a 3.5-fold (χ^2^ test, *P* < 2.2 × 10^−16^) increase of deletions relative to insertions in the mouse compared to the human lineage. It is consistent with the previously reported rodent lineage deletion bias (Laurie et al. 2012) and demonstrates that this bias applies to transcriptional regulatory sequence to at least the same extent as protein-coding sequence (Taylor et al. 2004).

We further classified those promoters that could be projected into orthologous sequence based on their transcriptional output in the opposing species (Fig. 1C). For example, conserved human promoters could show matched or divergent promoter activity (shown by the green arrow in Fig. 1C and promoter 1/2 in Fig. 1A) depending on whether they are expressed in the same tissues in human and mouse, diminished promoter activity (orange arrow), or no evidence for promoter activity (black cross) at the orthologous position in the mouse genome. Compared to conserved promoters with matched expression patterns, the more evolutionarily volatile promoters collectively show lower levels of expression (Mann–Whitney *U* tests, *P* < 2.2 × 10^−16^) (Fig. 1E). This suggests that relatively weakly expressed promoters are more likely to have been recently acquired or lost in evolution.

Consistent with our previous findings for human (Forrest et al. 2014), promoters with tissue-restricted expression (Fig. 1F) were significantly more likely to be diverged for promoter activity between species than those with broader expression (2.3-fold enrichment for mouse, 1.7-fold for human, χ^2^ *P* < 2.2 × 10^−16^). All modes of promoter turnover (insertion, deletion, and the gain or loss of expression at aligned sequences) contribute to the greater rate of tissue-restricted promoter birth and death (Fig. 1F; Supplemental Table 2; Supplemental Fig. 1). Relative to all promoters, tissue-restricted promoters show an enrichment of TATA box motifs, while broadly expressed and housekeeping promoters are enriched for CpG island overlap (Carninci et al. 2006). Both of these patterns are recapitulated by newly inserted promoters and hold true regardless of the promoter's evolutionary history or fate in another lineage (Supplemental Fig. 2).

If we consider the number of promoters that are biased in expression to a particular tissue, mouse and human tissues are highly correlated (Spearman's ρ = 0.79, *P* < 2.2 × 10^−16^) (Fig. 1F). Neuronal cells and tissues (but not glial astrocytes) consistently exhibit among the highest numbers of tissue-biased promoters. In agreement with previous observations (Khaitovich et al. 2005), they also show the highest fractions of matched promoters (Fig. 1F). Testis provides a striking counterpoint to the neuronal samples—while possessing similar high numbers of tissue-biased promoters, they exhibit the highest levels of promoter birth and death of all compared sample types.

The mouse and human lineages are highly correlated in the proportion of matched promoters expressed in each tissue (Spearman's ρ = 0.84, *P* < 2.2 × 10^−16^). This indicates that gene regulation in orthologous tissues has generally evolved synchronously along the diverging lineages.

### Functional promoter turnover in orthologous sequence

**Figure 2.**
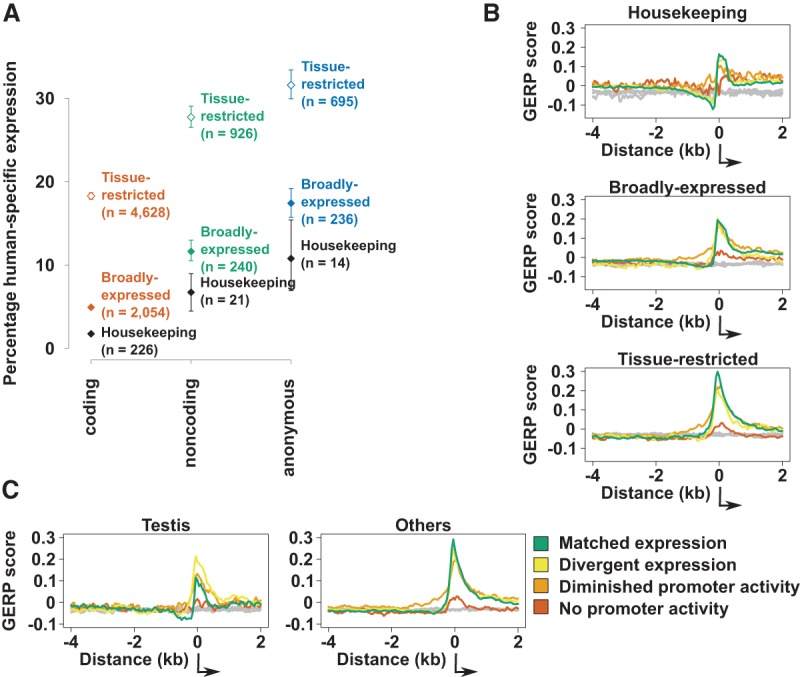
Expression turnover at aligned promoters. (*A*) The percentage of human promoters of a particular class and expression profile which can be aligned to mouse but show no transcriptional activity at the aligned position. The error bars represent the 95% confidence interval from 1000 samplings of the data with replacement. (*B*,*C*) Mean GERP conservation scores in 50-bp windows around human protein-coding promoters with different evolutionary outcomes. Gray lines indicate the GERP scores for genome permuted intervals. The standard error of these mean scores is negligible and not visible on this scale. The direction of transcription is shown by the black arrows. The sample sizes of promoters contributing to each line are detailed in Supplemental Table 3.

Of those human promoters that could be aligned to an orthologous sequence in the mouse, 18.2% showed no detectable transcriptional initiation in any of the 399 mouse FANTOM5 CAGE samples, while 23.3% showed no initiation in our 52 matched samples (correspondingly, 13.2% and 15.9% of mouse aligning promoters were not active in all human and our matched samples, respectively). The frequency of this expression turnover without sequence turnover is particularly high for tissue-restricted promoters (Fig. 2A). Beyond this, both noncoding and anonymous (see Supplemental Methods) promoters show an elevated rate of expression turnover relative to protein-coding promoters (Fig. 2A; Supplemental Fig. 3). These patterns persist throughout all tissue-biased promoters and are consistent across tissues (Supplemental Fig. 4).

For promoters with orthologous human:mouse sequences, expression turnover (Fig. 2B,C) was related to their associated GERP scores (Davydov et al. 2010). These scores are a measure of nucleotide substitution rate relative to a genome-wide expectation of neutral evolution, where values above zero indicate constraint relative to the neutral estimate. Whether the orthologous promoters exhibited matched, divergent, or diminished expression, the nucleotide substitution rate was similar over the core promoter (Fig. 2B) and significantly constrained. However, in the cases where promoter activity appears to be completely absent from one species, there is a dramatic reduction in sequence conservation (dark orange curve, Fig. 2B,C). This is a consistent finding regardless of the breadth or tissue bias in the expressing species but is more pronounced for human than mouse promoters (Supplemental Fig. 5). Diminished sequence conservation supports the notion that these lineage-specific promoters represent the birth and death of functional promoters within one of the lineages.

Housekeeping and testis-biased promoters both exhibit pronounced regions of negative GERP scores at the core promoter and immediately upstream (Fig. 2B,C; Supplemental Figs. 5, 6). Such scores, indicating a substitution rate that exceeds the expected neutral rate, could be interpreted as positive selection (Haygood et al. 2007). They can alternately be explained as locally elevated mutation rates (Taylor et al. 2006, 2008). As the negative GERP scores are most pronounced in the promoters that exhibit conserved rather than divergent expression, we interpret this as evidence for a locally elevated mutation rate in these regions.

### Frequent lineage-specific insertion and deletion of promoters

**Figure 3.**
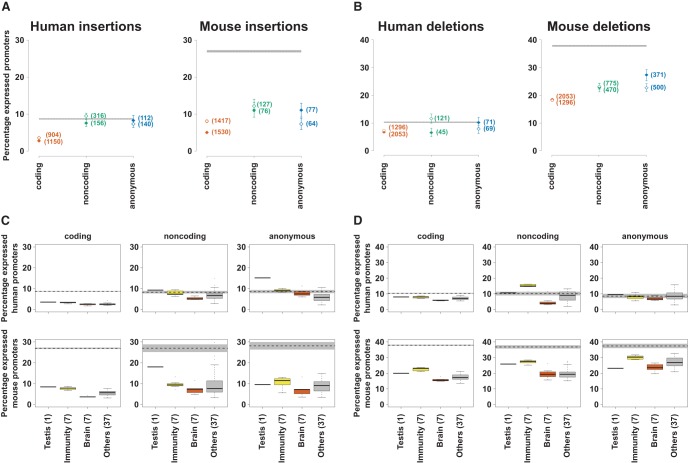
Recent promoter insertions and deletions in the human and mouse lineages. (*A*,*B*) The percentage of promoters of a particular class and expression profile which have been recently inserted (*A*) or deleted (*B*) in the human and mouse lineages. The closed diamonds represent broadly expressed promoters, while open diamonds show results for tissue-restricted promoters. The numbers of promoters in each category are shown in parentheses next to these points. The error bars represent the 95% confidence interval from 1000 samplings of the data with replacement. The gray bar shows the same 95% confidence interval for genome permuted intervals. The dashed line describes the mean of this expected distribution. (*C*,*D*) Percentage of promoters with tissue-biased expression that were inserted (*C*) or deleted (*D*), subdivided by biased tissue expression, where the number of samples for each tissue (described in Fig. 1F) is shown in parentheses. The gray bars show the 95% confidence interval for genome permuted intervals for each promoter class, while the dashed line shows the mean of this distribution.

To understand the types of promoters that tend to be subject to sequence turnover, we considered separately the evolutionary behavior of promoter classes showing different expression profiles. As with expression turnover, we observe increased turnover of noncoding and anonymous promoters but here broadly expressed and tissue-restricted promoters of a given class show more comparable frequencies of both insertions and deletions (Fig. 3A,B). It is interesting to note that, while noncoding and anonymous promoter insertions and deletions are similar to the genome-wide insertion and deletion rate in humans, they are substantially suppressed relative to the genomic background in the mouse lineage. This could indicate greater purifying selection in the mouse on noncoding and anonymous promoters, or represent different genomic biases in the occurrence of insertions and deletions between the two lineages.

There are clear differences in the rate of promoter insertion and deletion across tissues (Figs. 1, 3C,D). Testis- and immune-biased promoters generally show a greater proportion of both insertions and deletions than those with expression biased to brain and other tissues (as judged by Mann–Whitney *U* tests, *P* < 0.05). Brain-biased promoters also often showed significantly fewer insertions and deletions when compared to the “other” tissue category in both human and mouse (Fig. 3C,D).

### Selective constraint on promoters in the human population

Although we can measure the functional turnover of promoters and changes in gene expression between species, it does not demonstrate that those altered promoter activities have biologically important consequences for the organism. The frequency distribution of derived alleles can be used to explore selective effects within a single lineage (Fay et al. 2001), where purifying selection will act to reduce the population frequency of deleterious alleles and diversifying selection will tend to elevate the frequency of new beneficial alleles. Unlike substitution rate estimates (Fig. 2B,C; Supplemental Figs. 5, 6), allele frequency distributions should only be confounded by implausibly extreme mutational heterogeneity that results in recurrent mutations to the same site within the divergence time of the measured population. With the recent availability of high-quality, uniform coverage, whole-genome sequences from a large, single population cohort (Gudbjartsson et al. 2015), it is now possible to perform derived allele frequency (DAF) tests to compare selection between arbitrary collections of human genomic intervals.

**Figure 4.**
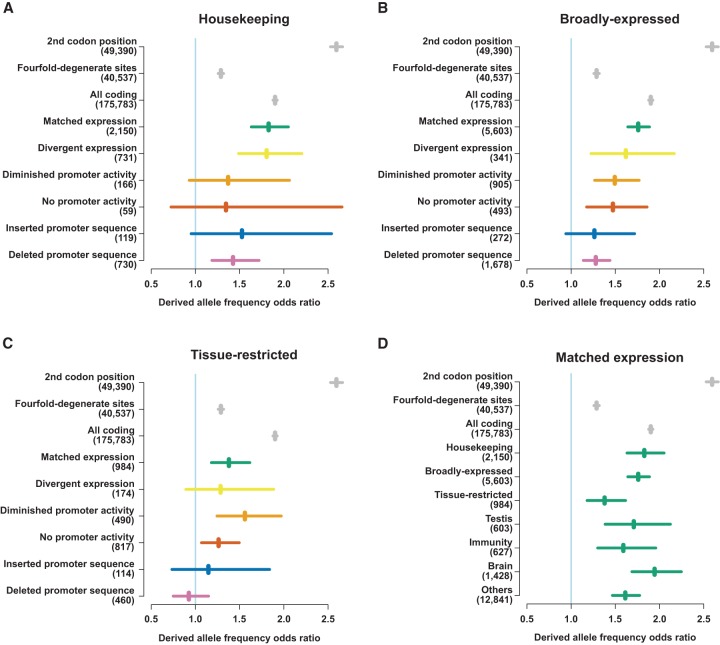
Derived allele frequencies in promoters of different evolutionary outcomes. (*A*–*C*) Odds ratios of derived allele frequencies for rare (<1.5%) and nonrare (>5%) derived alleles compared between the genome-wide distribution and the tested sequence category as labeled. Odds ratios of 1.0 indicate equality with the genome-wide distribution, higher values indicate relative selective constraint, and values <1 are indicative of net positive selection. Odds ratios for single nucleotide polymorphisms (SNPs) at the 2nd codon position, fourfold-degenerate sites and within all protein-coding sequence are shown in gray as points of reference for comparison. The numbers of informative SNPs overlapping each category are shown in parentheses next to the axis labels. (*D*) Derived allele frequency odds ratios for promoters with matched expression between species and different expression profiles and tissue biases. As in *A*–*C*, odds ratios for SNPs at the 2nd codon position, fourfold-degenerate sites, and within all protein-coding sequence are shown in in gray. The numbers of SNPs overlapping each category are shown in parentheses next to the axis labels.

Applying DAF tests using the genome-wide allele frequency distribution as a proxy for neutral evolution (fourfold-degenerate protein-coding sites are constrained relative to the bulk genome) (Fig. 4), we find that promoters with conserved patterns of housekeeping and broad expression exhibit significant constraint, at a similar level to that seen for protein-coding sequences (Fig. 4A,B,D). By DAF measures, promoters with conserved tissue-restricted expression tend to exhibit less constraint than those with conserved broad expression (Fig. 4D), which is the opposite pattern to that from substitution rate-based estimates (Fig. 2B; Supplemental Fig. 5). A similar discrepancy is seen for promoters with testis-biased expression, where DAF measures show they are similarly constrained to promoters with other tissue expression biases (Fig. 4D), but they also appear to exhibit higher rates of nucleotide substitution (Fig. 2C; Supplemental Fig. 6). These observations are consistent with our prior conclusion that testis-biased promoters and those with housekeeping-like expression exhibit locally elevated nucleotide substitution mutation rates. Notably, each of these promoter categories is likely to be active in the germline.

All categories of promoter that have measurably diverged between human and mouse tend toward lower levels of constraint than those with conserved expression. This is particularly so for those where the promoter-containing sequence has been inserted in the human lineage or deleted from the mouse, both of which exhibit estimates of selection that overlap the expectation of neutral evolution (Fig. 4; Supplemental Fig. 7). These findings highlight the possibility that many of the observed changes in transcriptional regulation, although measurable at the molecular level, may be invisible to selection on the organism level.

### The gain of tissue-restricted promoters driven by transposable element insertion

**Figure 5.**
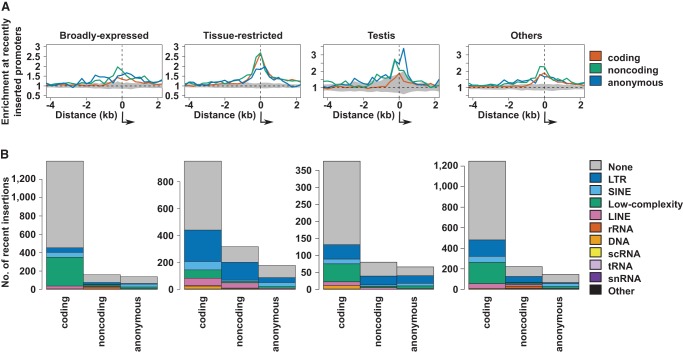
Promoter insertions frequently contain repetitive elements. (*A*) Enrichment of repetitive elements across recently inserted human promoters relative to the genome-wide expectation for insertions across promoter classes and expression profiles. The 95% confidence interval for genome permuted intervals is shown in gray, and the direction of transcription is shown by the arrows. The numbers of promoters which contribute to each enrichment are shown in the corresponding histograms in *B*. (*B*) Frequency of repetitive element families across recently inserted human promoters of the expression profiles, as in *A*.

It has previously been suggested that transposable element insertions can be responsible for the de novo birth of noncoding RNA (ncRNA) genes (Cao et al. 2006). Our genome-wide data on insertions revealed that this is a general phenomenon driving promoter insertion for both coding and noncoding transcripts (Fig. 5). Comparing repetitive element density (see Supplemental Methods) between recently inserted and conserved promoters, we find that the newly inserted are 4.9- and 3.7-fold enriched in human and mouse, respectively, across all categories of repeat (χ^2^, *P* < 2.2 × 10^−16^), and similar enrichments are found across all promoter types and tissue categories (Fig. 5; Supplemental Table 4). As transposable elements are expected to be enriched in recently inserted sequence, we also compared the frequency of repetitive elements at recently inserted promoters to the genome-wide background frequency for repetitive elements in recently inserted sequence. Repetitive elements are significantly enriched at all newly inserted promoters even by this more stringent measure (Fig. 5A). This finding applies to recently inserted promoters of all classes and most tissue-biased expression patterns (Fig. 5A; Supplemental Figs. 8, 9).

Low complexity repeats are common at recently inserted broadly expressed protein-coding promoters (at least 3.4-fold enriched, maximum Mann–Whitney *U* tests, *P* = 5.3 × 10^−5^ relative to tissue-restricted promoters). Long terminal repeats (LTRs) are most frequent at novel tissue-biased promoters (at least 2.9-fold enriched, maximum Mann–Whitney *U* tests, *P* = 6.2 × 10^−4^ relative to broadly expressed promoters; mouse anonymous promoters were nonsignificant, *P* = 0.2) (Fig. 5B; Supplemental Figs. 10, 11). LTRs were particularly prominent at inserted promoters showing testis- and other-biased expression relative to those showing immunity- and brain-biased expression (Mann–Whitney *U* tests, *P* < 0.05).

We observed no corresponding enrichment of repetitive elements at recently deleted promoters (Supplemental Fig. 12). We also repeated this analysis, considering only simple and satellite repeats as these elements are prone to deletion (Usdin 2008) but were unable to detect any clear enrichments around either recently inserted or deleted promoters.

### Compensatory promoter turnover

Thus far, we have considered the evolution of individual promoters. We now turn to protein-coding genes as the unit of study. As the majority of transcriptional units have multiple promoters (Carninci et al. 2005), the existence of additional promoters for a protein-coding gene provides some redundancy and conceptually an easier path to promoter turnover. For this, we focused on a set of 15,768 protein-coding gene-pairs defined as being 1:1 orthologs between human and mouse, of which 13,881 (88.0%) and 13,126 (83.2%) were associated with at least one expressed promoter in human and mouse, respectively.

**Figure 6.**
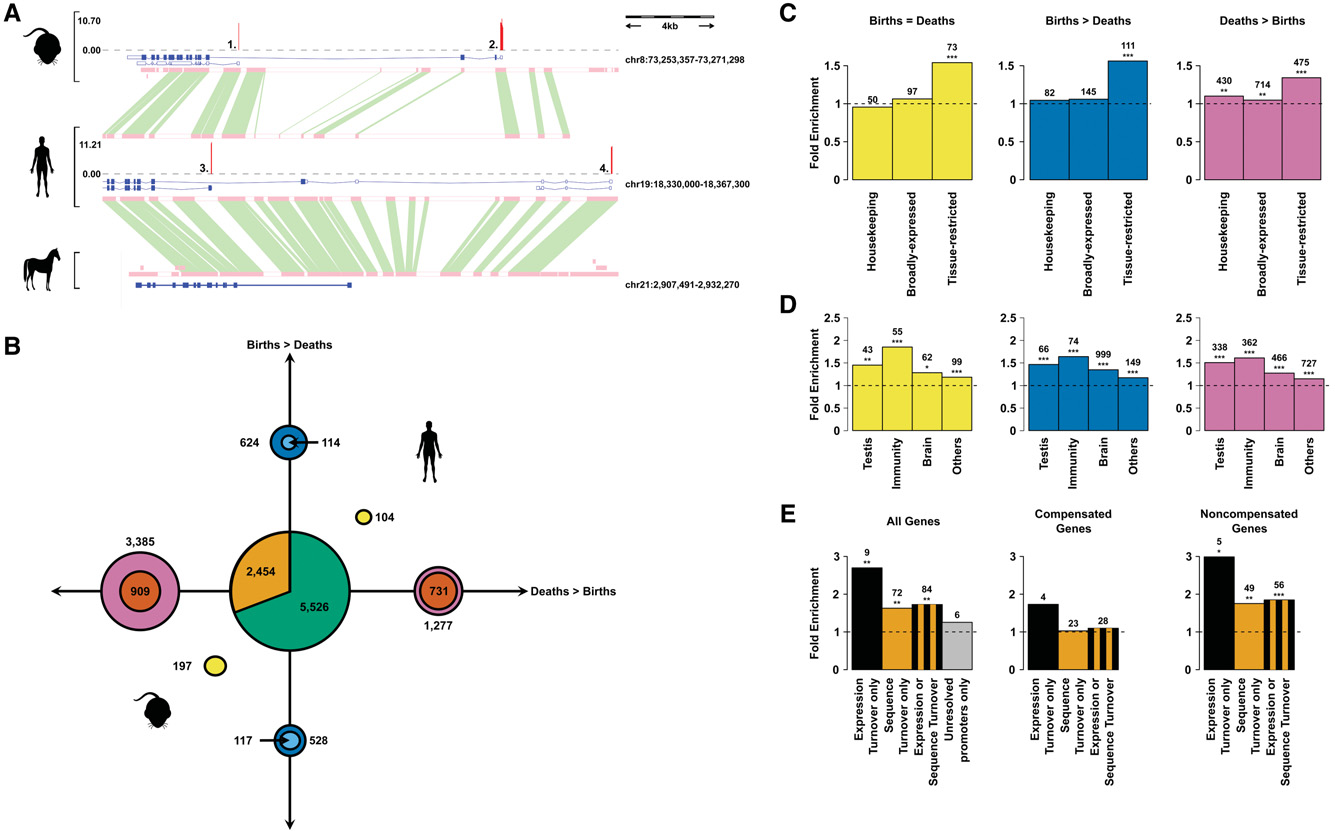
Compensatory promoter turnover and positive selection. (*A*) Human, mouse, and horse alignments at the *PDE4C* locus. Four promoters are shown, which are conserved (promoters 1 and 3), human-deleted (promoter 2), or mouse-deleted (promoter 4). Gene models supported by the CAGE promoters are shown in the blue boxes, where solid boxes represent coding exons and empty boxes noncoding exons. The histograms in red describe the log_2_-transformed expression level of the annotated promoters. Orthologous sequence identified between species is highlighted by the green boxes between these sequences. (*B*) Frequencies of 1:1 orthologous genes in human and mouse categorized by the type of promoter sequence turnover events. The blue circles represent genes with a greater proportion of promoter births than deaths, while the purple circles similarly represent genes with a greater proportion of promoter deaths. Genes with an equal number of promoter births and deaths are shown in the yellow circles. All genes are shown in the outer circles, while the numbers in the inner circles shows those with evidence for compensatory promoter turnovers. Genes with only expression turnover at their promoters are shown in the orange segment, while the remainder of the green circle indicates the number of genes with a conserved promoter architecture (*C*,*D*). Enrichments of human orthologous genes with different turnover events and expression profiles relative to genes with a conserved promoter architecture. χ^2^ test, (*) *P* < 0.05, (**) *P* < 0.01, (***) *P* < 0.001. (*E*) Enrichments of orthologous genes with coding sequence positive selection. Genes are classified by the possible different evolutionary outcomes of their associated promoters relative to genes with a conserved promoter architecture. χ^2^ test, (*) *P* < 0.05, (**) *P* < 0.01, (***) *P* < 0.001.

For each gene, we collated the associated promoters in both human and mouse (e.g., *PDE4C*) (Fig. 6A) and asked if all promoters could be aligned in both species (ignoring promoters with ambiguously resolved alignments). Of the genes tested, 7980 (58.4%) exhibited a perfectly conserved complement of aligned promoter sequences. Of the remaining genes, there were few with a simple compensatory architecture where the number of gains equals the number of losses in a lineage, but many genes showing evidence for compensatory turnovers where the total number of divergent promoters was less than the sum of diverging promoters in each species (see Methods; Fig. 6B). Inserted and deleted promoters had little effect on the inferred length distribution of 5′ UTRs. In contrast, promoters whose sequence aligns but exhibit discordant activity between species are associated with longer 5′ UTRs (Mann–Whitney *U* tests, *P* < 1.1 × 10^−3^) (Supplemental Fig. 13), a pattern that is consistent in both human and mouse. Promoters found at genes with only one promoter generally showed a higher level of expression than those at genes with multiple promoters (Supplemental Fig. 14), regardless of their evolutionary fate.

We found a clear bias for the genes with promoter turnover events to show tissue-biased expression (Fig. 6C,D; Supplemental Fig. 15). This bias was consistently most evident for immunity-biased expression but not restricted to any particular tissue (Fig. 6D) and was even seen for genes with brain-biased expression that by other measures have appeared to be relatively conserved.

### Promoter turnover and adaptive evolution

To explore functional biases in the types of genes subject to promoter gain and loss events, we performed Gene Ontology (GO) term enrichment analysis. Mouse lineage-specific insertions were enriched for immune system-related terms such as “defense response to other organism” (odds ratio 2.77, *P* = 4.13 × 10^−7^, FDR = 4.97 × 10^−3^), whereas human-specific insertions and deletions did not show any enrichments that met our 1% false discovery rate threshold. Genes with at least one conserved promoter and those with promoters of unresolvable evolutionary history did not show any significant GO term enrichments, even at the less stringent threshold of *P* < 0.001. Unlike sequence turnover events, in our current data, expression turnover events cannot be assigned to a specific lineage, so these were considered collectively for the human and mouse lineages. GO terms related to blood coagulation, response to external stimulus, and cell junction assembly were all significantly enriched (*P* < 4.89 × 10^−6^, FDR < 7.5 × 10^−3^) but with modest odds ratios of 1.2 to 1.3 (Supplemental Table 5).

High rates of promoter turnover in immune tissues (Figs. 1F, 3C,D, 6D) and the significant enrichment of genes involved in host immune defense responses suggest a role for promoter turnover in adaptive evolution. Compared to genes with conserved promoter architectures (no gains or losses), genes that have experienced promoter or sequence turnover are more likely to have also experienced positive selection (Kosiol et al. 2008) acting on their coding sequence (1.7-fold enrichment, χ^2^ *P* = 2.0 × 10^−4^) (Fig. 6E). This enrichment was specific to genes not showing the compensatory turnovers described above, implying that a change to promoter number accompanies regulatory adaptation. Enrichment for positive selection was found across the majority of tissue expression biases but was most evident in genes with sequence turnover and where expression was biased toward immune, testis, and brain expression (Supplemental Fig. 16).

When genes duplicate into paralogous copies within the same genome, they may initially exhibit functional redundancy, which provides an opportunity for subfunctionalization (splitting of ancestral functions between copies) or neofunctionalization (the acquisition of new functions while the other copy retains the original functions) (Long et al. 2003). Consistent with this, we find that genes with paralogs are significantly more prone to promoter turnover than genes without paralogs (1.1-fold, χ^2^ *P* = 5.0 × 10^−12^). Similarly, genes in large gene families (more than 10 members) show a significantly elevated rate of promoter turnover (1.2-fold increase in both human and mouse, χ^2^ *P* < 3.0 × 10^−22^). Considering just the promoter insertions and deletions where we can resolve gains from losses and the lineage of the change, the human lineage genes with paralogs show a 1.4-fold excess of insertions over deletions (χ^2^, *P* = 1.5 × 10^−3^) and those in large gene families show a 1.7-fold excess (χ^2^, *P* = 4.9 × 10^−9^), which may indicate a predominance of neofunctionalization. In contrast, genes with multiple copies in the mouse lineage show no bias toward insertions or deletions (χ^2^ *P* = 0.89 and 0.23 for paralogous and gene family analyses, respectively).

The apparent coupling of adaptation at the levels of protein sequence and transcription regulation along with the excess promoter turnover at genes with paralogous copies suggest that the rewiring of transcriptional regulation through the birth and death of functional promoters has contributed to the adaptive diversification of humans and mice from a common ancestor.

## Discussion

We have performed a comprehensive study of several modes of promoter evolution: (1) divergence of expression pattern; (2) the gain or loss of promoter activity in conserved sequence; and (3) the insertion and (4) deletion of promoter-containing sequence. The comparison between lineages is based on 52 matched tissue and cell samples, but for some analyses, it also depends on genomic annotation to assign promoters to genes which, although high quality in both focal species, is more comprehensive for humans than mice. Despite this imbalance, our results are strikingly consistent between lineages, and the main difference, an increased promoter deletion rate in mouse, is independent of gene assignment. We conservatively estimate that 14,072 human and 18,016 mouse lineage promoter births and deaths have occurred since these species diverged from a common ancestor around 100 million years ago (Murphy et al. 2007). At least 41.6% of protein-coding genes have experienced promoter sequence gain or loss. With the inclusion of expression turnover, this number increases to 56.7%. These observations demonstrate that both the birth and death of functional promoters represent major mechanisms of transcriptional regulatory evolution in mammals.

This is not to diminish the potential role of enhancer gain and loss in regulatory evolution, as putative enhancers are gained and lost throughout mammalian evolution at an even higher rate than putative promoters in liver tissue (Villar et al. 2015). Measuring active enhancers across multiple matched tissues as we have done for functional promoters may reveal an even more dynamic regulatory landscape of the mammalian genome than we currently appreciate.

The functional consequences of promoter gain and loss potentially extend beyond the level and pattern of transcript expression. This can manifest as the differential inclusion of regulatory sequences, including miRNA binding sites, or the alteration of an encoded N-terminal amino acid sequence, as is the case for *PDE4C* (Fig. 6A), an example that has previously been noted (Johnson et al. 2010) and a type of change that is a recurrent evolutionary feature of the PDE4 gene family.

Compared to promoters with broad patterns of activity, those with more tissue-specific expression tend to exhibit higher nucleotide substitution constraint but are more rapidly evolving by all other measures. Both protein-coding sequences and *cis*-regulatory elements that are restricted in activity have similarly been found to be more rapidly evolving than those active in a broader range of tissues (Brawand et al. 2011; Cheng et al. 2014), an observation that is possibly explained in terms of greater pleiotropic constraints with broad expression. While pleiotropic constraints are an attractive model with which to explain the clear relationship between breadth of expression and evolutionary volatility, the pattern of expression is highly correlated with promoter architecture (Carninci et al. 2006), and expression level is also a strong predictor of evolutionary behavior.

Although we observe considerable heterogeneity of promoter turnover between tissues and cell types, it is remarkable how consistent these patterns are between humans and mice (Fig. 1F). This suggests that the same biological systems are similarly inclined to the modification of transcriptional regulation in both lineages. For example, testis and immune tissues are frequently found to exhibit evidence for adaptive evolution in protein-coding sequence (Kosiol et al. 2008) and differences in gene expression between species (Yue et al. 2014). Promoters with expression biased to these tissues generally show the greatest rates of insertion and deletion in both lineages (Fig. 3). The role for promoter birth and death in adaptive evolution is further supported by the significant enrichment of positively selected protein-coding sequences in those genes that have experienced sequence or expression turnover (Fig. 6E). Negative GERP scores upstream of housekeeping and testis promoters but high levels of constraint measured by DAF tests are all consistent with the suggestion that promoter regions active in the germline have elevated nucleotide substitution mutation rates (Taylor et al. 2006, 2008), an observation that may relate to elevated replication-associated mutation rates around the binding sites of some transcription factors (Reijns et al. 2015). This suggestion is further bolstered by the observation that promoters with conserved testis-biased expression have more negative GERP scores than those with divergent testis-biased expression.

By all measures of promoter evolution (insertion, deletion, nucleotide substitution, and expression turnover), nervous tissues and cell types showed the highest conservation between species and also the greatest constraint within the human population. Slow evolution of nervous tissue gene expression (Brawand et al. 2011; He et al. 2014) is in agreement with the higher protein-coding sequence conservation for genes expressed in the brain (Khaitovich et al. 2005). This high level of conservation in neuronal regulation and protein-coding sequence may be considered surprising, as anatomically and metabolically the brain appears to be one of the most diverged organs between humans and mice (Somel et al. 2013; Bozek et al. 2014). The exceptional transcriptional diversity of the brain (Fig. 1F), matched only by the testis, could go some way to reconciling the perceived organ divergence with molecular conservation. Although proportionally the brain promoters are most conserved, it has the highest count (*n* = 6623) of tissue-biased promoter turnover.

The principal difference in promoter evolution between lineages is the large excess of promoter deletions in the mouse compared with either the insertion rate in mouse or the deletion rate in the human lineage (Fig. 1D). This is consistent with reports of genome-wide excess deletions early in the rodent lineage (Laurie et al. 2012). The deletion excess in rodents may also explain more subtle differences between human and mouse promoter evolution. We propose that an ancestral promoter that is no longer maintained by purifying selection is most likely to be deleted in the rodent lineage but to diminish, and eventually lose, promoter activity in the human lineage. This hypothesis is supported by differences between lineages in the sequence conservation of promoters with diminished activity (Fig. 2; Supplemental Figs. 5, 6) and the reduced constraint demonstrated by the human derived allele frequency tests for human promoters that have been deleted from the mouse genome (Fig. 4; Supplemental Fig. 7). The implication of this is that, compared to the mouse, the human genome may contain many weakly transcriptionally active, but selectively invisible, promoters.

The insertion of novel promoter-containing sequences is associated with transposable elements, particularly those containing LTRs. While there have been reports of repetitive elements in species-specific regulatory DNA (Vierstra et al. 2014; Yue et al. 2014), we show for the first time that they are preferentially associated with inserted, rather than deleted, promoter sequences (Fig. 5; Supplemental Figs. 8, 9, 12). This suggests that ancestral LTR-derived promoters are either stably exapted into both the human and mouse lineages, or probably more frequently, they have been lost from both lineages. Such transposable elements have previously been found to acquire host genome functions (Bejerano et al. 2006) and selective constraint (Lowe et al. 2007; Lowe and Haussler 2012). LTRs have also been seen to exhibit tissue-restricted expression (Faulkner et al. 2009; Fort et al. 2014), so they were a priori good candidates for novel genic, tissue-restricted promoters. Despite the important repeat element contribution to promoter birth, the majority of promoter-containing sequence insertions were not associated with repetitive elements. It will be interesting to explore in more detail the origin of these novel promoters, which in many cases do have identifiably homologous sequence located elsewhere in the genome.

The gain and loss of functional promoters is a major contributor to the evolution of transcriptional regulation in mammals. Distal enhancer elements are known to confer regulated domains of restricted expression on specific genes (Visel et al. 2007; Anderson and Hill 2014) and show extraordinary turnover across mammalian species (Odom et al. 2007; Ballester et al. 2014). Here, we have observed that evolutionarily volatile promoters similarly show tissue-biased activity. As most protein-coding genes have multiple promoters (Carninci et al. 2005) and are expressed in multiple tissues (Su et al. 2004), the turnover of functional promoters may thus represent another important but previously under-appreciated mechanism for the evolution of modular transcriptional divergence.

## Methods

### Promoter definition

We considered only CAGE tag clusters that were predicted as genuine transcriptional start sites using a strict sequence classifier cut-off (Forrest et al. 2014); these clusters are referred to as promoters throughout this work. CAGE tags supporting orthologous and paralogous genes were identified using the Ensembl 67 (May 2012) build (Flicek et al. 2014).

TATA boxes were identified on either DNA strand in the 20- to 30-nt window upstream of promoters using the RSAT pattern matching tool (Turatsinze et al. 2008) with a minimum *P*-value threshold of 1 × 10^−3^. All other parameters were left at their defaults. CpG island-promoters were defined as those with any overlap with CpG island locations extracted from the UCSC Genome Browser (Kent et al. 2002).

### Expression data

We created a list of 52 cell and tissue samples matched between human and mouse with CAGE expression data available in both species. These samples were further labeled as “testis,” “immunity,” or “brain,” depending on their tissue of origin (Supplemental Table 1). Astrocyte samples, which appeared to be outliers relative to the other brain samples, and all other samples were labeled as “others.” For each promoter, we calculated a single tags-per-million count (TPM) expression level as the mean of relative log expression (RLE)-normalized TPM values from all biological replicates available for each sample.

Promoters were defined as “broadly expressed” or “tissue-restricted” as in Forrest et al. (2014). Broadly expressed promoters were recorded as being biased in a particular sample if the mean TPM in that tissue type was at least five times the median TPM across all tissue types for that promoter (Marques and Ponting 2009). Tissue-restricted promoters (with a median TPM of 0) were identified as biased if their mean TPM for a given tissue type was >1.

### Multispecies alignments

Promoter sequences were projected into six mammalian species (human, mouse, dog, horse, cow, pig) from the 12-way mammalian EPO alignments (May 2012 release) from Ensembl (Flicek et al. 2014) and from pairwise genome alignments released by UCSC (Kent et al. 2002). A full description of our processing of these alignments can be found in Supplemental Methods.

In brief, we combined both sets of alignments to increase our power to detect an aligned position and thereby increase our confidence that alignment gaps genuinely arise from unaligned sequence. Recently inserted promoters are defined as being within gapped, unaligned, or unmapped sequence in all four outgroup species (dog, horse, cow, and pig) and the opposing species. Human promoters that were aligned (but not projected into multiple locations) in at least one outgroup species but not found in the mouse genome were defined as recently deleted mouse promoters. Similarly, mouse promoters within gapped, unaligned, or unmapped sequence in human but aligned in at least one outgroup species were defined as recently deleted human promoters. Finally, 14,400 (18.8%) and 11,966 (23.2%) promoters in human and mouse, respectively, remain unclassified where the whole-genome multiple species alignments and pairwise species alignments gave differing results. Human and mouse promoters and their evolutionary outcomes are reported in Supplemental Files 1 and 2, respectively.

Deviations from genome-wide expectations were assessed by randomly permuting the position of all promoter sequences within regions containing uniquely mappable 36mers (intervals with a mappability score of 1 from the CRG 36mer alignability tracks) while avoiding the ENCODE DAC blacklisted regions (The ENCODE Project Consortium 2012).

### Compensatory turnover

Compensatory turnover (*T*_c_) was calculated following the method applied by Mustonen et al. (2008). For a gene, the absolute difference in functional promoter count between human *P*_*h*_ and mouse *P*_*m*_ is subtracted from the number promoter of gain or loss events (*t*) in that gene between the two species.

$$T_{c}=\sum t-|P_{h}-P_{m}|$$

A gene is considered subject to compensatory turnover when *T*_*c*_ > 0. For the results presented, this calculation considered sequence turnover and complete expression turnover (zero expression in one species) but not diminished expression.

### Derived allele frequency tests

Aggregate polymorphism data including observed allele frequencies from the whole genome (median 20× coverage) sequencing of 2636 Icelandic individuals (Gudbjartsson et al. 2015) was obtained from the European Variant Archive (accession: PRJEB8636). Variants were resolved into ancestral and derived alleles through reference to the Ensembl human ancestor reconstructed sequence based on the 12-way mammalian EPO alignments (as used for between-species analysis). Variants with unresolved ancestral states and non-single-nucleotide polymorphisms were discarded. Polymorphisms were split into rare (<1.5%) and nonrare (>5%) derived allele frequency categories. Counts of polymorphic sites in the rare and nonrare categories were compared in a Fisher's exact test to the rare:nonrare polymorphic site count ratio for the whole genome. Fisher's exact test was performed using the *fisher.test* function in R (R Core Team 2015; version 3.0.0) which provides a *P*-value and 95% confidence intervals in addition to the odds ratio. The 1.5%-threshold was defined based on qualitatively maximizing the odds ratio while minimizing the confidence interval for a comparison of second codon positions (assumed to be constrained, as changes at these sites always alter the encoded amino acid) and fourfold-degenerate sites (a proxy for neutral evolution) in protein-coding sequence (Fig. 4).

### Positive selection

Positively selected protein-coding genes were collected from the “Pos Sel Genes” track (Kosiol et al. 2008) for the hg18 genome assembly in the UCSC Genome Browser. Genes subjected to positive selection were identified as any which passed any of the likelihood ratio tests with an FDR < 0.05 described in Kosiol et al. (2008).
